# Retinal Artery Occlusion and Associated Risk of Cerebrovascular Disease Related Hospitalization: A National Inpatient Study

**DOI:** 10.7759/cureus.27354

**Published:** 2022-07-27

**Authors:** Manpreet Kaur, Samreen Ahmed, Hadia Younis, Sanobar Jaka, Anusheel ., Johanna S Canenguez Benitez, Nikhita S Roshan, Ninad Desai

**Affiliations:** 1 Medicine, Sri Guru Ram Das Institute of Medical Sciences and Research, Amritsar, IND; 2 Neurology, University of Illinois at Chicago, Chicago, USA; 3 Medicine, Peshawar Medical College, Peshawar, PAK; 4 School of Global Public Health, New York University, New York, USA; 5 Medicine, Shanti Gopal Hospital, Ghaziabad, IND; 6 Internal Medicine, Larkin Community Hospital, South Miami, USA; 7 Neurology, Father Muller Medical College, Mangalore, IND; 8 Neurology, St. Vincent's Medical Center, Bridgeport, USA

**Keywords:** diabetes and cvd, prevalence rate, hospitalization outcomes, cerebro vascular disease, central retinal artery occlusion

## Abstract

Objectives

To evaluate the demographic and comorbid risk factors for cerebrovascular disease (CVD) hospitalization in patients with retinal artery occlusion (RAO) and study the impact on hospitalization outcomes.

Methods

We conducted a retrospective cross-sectional study using the Nationwide Inpatient Sample (NIS, 2019). We included 62,255 adults (age 18-65 years) with the primary diagnosis of CVD. The study sample was divided by the co-diagnosis of RAO (N=1,700). A logistic regression model was used to evaluate the odds ratio (OR) of association for risk factors leading to CVD hospitalization in patients with RAO, with the non-RAO cohort as the reference category.

Results

The majority of the CVD patients with RAO were elderly (51-65 years, 68%), females (54%), and whites (47%). Yet, demographics did not significantly impact the association with CVD hospitalization between RAO and non-RAO patients. There was a significant difference in the geographic distribution of CVD hospitalizations with RAO, with the highest prevalence in the East North Central Atlantic (21%) and South Atlantic (18%) regions, and the lowest in the Mountain (4%) and East South Central (4%) regions. Comorbid diabetes with complications (69%), and complicated hypertension (55%) were most prevalent in patients with RAO thereby increasing the risk for CVD hospitalization by 7.8 (95% CI 6.9-8.8) and 1.8 times (95% CI 1.6-1.9), respectively. Patients with RAO and having major severity of illness were at increased risk of CVD hospitalization (OR 2.8, 95% CI 1.9-3.9). Patients with RAO had a significant difference in adverse disposition, including transfer to the skilled nursing facility (SNF)/intermediate care facility (ICF) (32% vs. 24%) and requiring home health care (16% vs. 11%) compared to non-RAO patients.

Conclusion

The prevalence of RAO in CVD hospitalization was 2.7%, and demographics did not have any impact on the increasing risk of CVD. Comorbid diabetes (by 685%) and hypertension (by 78%) potentially increase the risk of CVD hospitalization in patients with RAO. These patients have a major severity of illness, leading to an adverse disposition. This calls for a collaborative care model to improve the quality of life in these at-risk patients with RAO.

## Introduction

Cerebrovascular disease (CVD) is the fifth leading cause of death and one of the leading causes of disability in the United States (US) despite a steady decline in stroke incidence since 2008 [1,2]. Improved primary prevention, attributed to better control of vascular risk factors and the use of statins in American adults, has contributed to this change, particularly in the white population. Additionally, there has been improved awareness of better blood pressure management and increased promotion of smoking cessation. A lesser degree of impact in declining these rates is early identification and management of an episode of stroke [2]. Despite that, as a consequence of the obesity epidemic, rates of diabetes and hypertension may increase, leading to an estimate that an additional 27 million people will have hypertension, eight million will have cardiovascular disease, and four million will suffer from the cerebrovascular disease [2].

CVD is broadly classified into ischemic and hemorrhagic strokes; ischemic stroke is caused by deficient blood and oxygen supply to the brain, whereas hemorrhagic stroke is due to bleeding or leaky blood vessels. About 85% of these cerebrovascular accidents are caused by ischemic events [1].

Central retinal artery occlusion (RAO) and CVD share a common pathophysiology mechanism of thromboembolism that interferes with blood and oxygen supply to the brain and retina, causing tissue hypoxia, which leads to infarction of brain tissues and retina if not tended urgently [3]. RAO leads to permanent vision loss and is considered an ocular emergency [4]. CVD and RAO share common risk factors such as high blood pressure, diabetes mellitus, hyperlipidemia, cardiovascular diseases, sedentary lifestyle, atrial fibrillation, smoking, and alcohol consumption [3,4]. The incidence of RAO is estimated at 0.85 per 100,000 per year, with a ten-year cumulative incidence of retinal emboli of 1.5%. RAO affects men slightly more frequently than women. The mean age of retinal artery occlusion is in the early seventies of life, although a few cases have been reported in patients younger than 30 years of age [5]. RAO was associated with an increased risk of stroke (a hazard ratio of 1.78) as per the data collected from the Korean national health insurance service that comprised 1,025,340 subjects [4]. After the occurrence of RAO, the risk of a subsequent vascular event is high, particularly an ipsilateral stroke within one month [6]. Ischemic strokes and other vascular events occurred in 8.6% and 9.9% of patients during their one-year follow-up interval [7]. A metanalysis by Zhou et al. found that patients who suffered from RAO had twice the increased risk of suffering from CVD. Both types, central RAO (CRAO) and branched RAO (BRAO), are associated with a significantly increased risk of cerebrovascular disease [8].

In this study, we would like to delineate demographic characteristics and hospitalization outcomes, including length of stay (LOS) and cost of care, the severity of illness, and disposition status in CVD inpatients with versus without RAO. Next, we will measure the predictive risk factors for CVD-related hospitalization in patients with RAO.

## Materials and methods

Study sample

We conducted a cross-sectional study using the nationwide inpatient sample (NIS, 2019), which is the largest inpatient database in the US, covering more than 4,400 non-federal community hospitals across 48 states and the District of Columbia [9]. As the NIS is publicly available de-identified data, it does not require approval from an institutional review board according to the agency for healthcare research and quality (AHRQ) and the department of health and human services [9].

We included 62,255 adult inpatients (age 18-65 years) hospitalized on a non-elective admission basis with a primary diagnosis of CVD, including middle cerebral artery syndrome, anterior cerebral artery syndrome, posterior cerebral artery syndrome, brain stem stroke syndrome, cerebellar stroke syndrome, pure motor lacunar syndrome, and/or pure sensory lacunar syndrome. The study sample was divided by the presence of a co-diagnosis of RAO (transient, central, and partial).

Variables

In this study, the variables of interest included demographic characteristics (age, sex, and race) and comorbidities, which are the co-diagnoses in the patient records, and we included metastatic cancer, diabetes with complications, hypertension (complicated), obesity, drug abuse, and peripheral vascular diseases (PVD). We included geographical areas and in the NIS they are based on the nine US Census Bureau, i.e., New England (Maine, New Hampshire, Vermont, Massachusetts, Rhode Island, Connecticut), Middle Atlantic (New York, Pennsylvania, New Jersey), East North Central (Wisconsin, Michigan, Illinois, Indiana, Ohio), West North Central (Missouri, North Dakota, South Dakota, Nebraska, Kansas, Minnesota, Iowa), South Atlantic (Delaware, Maryland, District of Columbia, Virginia, West Virginia, North Carolina, South Carolina, Georgia, Florida), East South Central (Kentucky, Tennessee, Mississippi, Alabama), West South Central (Oklahoma, Texas, Arkansas, Louisiana), Mountain (Idaho, Montana, Wyoming, Nevada, Utah, Colorado, Arizona, New Mexico), and the Pacific (Alaska, Washington, Oregon, California, Hawaii) [9].

The hospitalization outcomes of interest included: severity of illness, LOS, total charges, disposition, and in-hospital mortality (all-cause). The severity of illness was based on the all-patient refined drugs (APR-DRGs) evaluated by 3M health information systems in the NIS. Disposition of the patient at discharge was classified as routine, transfer to short-term hospitals, transfers to other facilities including skilled nursing facilities (SNF), intermediate care facilities (ICF), home health care, and discharge against medical advice [9].

Statistical analysis

We used Pearson's chi-square test and an independent-sample t-test for categorical data and continuous data (LOS and total charges), respectively. Descriptive statistics were used to delineate the differences in demographics and hospitalization outcomes in CVD inpatients without RAO vs. with RAO. A binomial logistic regression model was used to evaluate the odds ratio (OR) of the association for RAO in CVD inpatients and was compared to the non-RAO cohort (as a reference category). A P-value <0.01 was used to determine the statistical significance and all statistical analyses were conducted using the statistical package for the social sciences (SPSS) version 27 (IBM Corp., Armonk, NY).

## Results

We included 62,255 patients who were hospitalized for CVD and the majority of them were elderly (51-65 years, 65%), females (56.1%), and whites (54.1%). The prevalence of RAO in the CVD inpatients was 2.73% (N = 1,700). A higher proportion of CVD inpatients with RAO were elders (51-65 years, 67.6%) and females (54.4%). There existed statistically no significant difference between RAO and non-RAO cohorts by sex (P = 0.146). While comparing the cohorts by race/ethnicities, there existed a significantly higher difference in blacks (30% vs. 25.8%) and hispanics (13.8% vs. 12%). Diabetes with complications (68.5% vs. 18.9%) was most prevalent in the RAO cohort followed by complicated hypertension (55.3% vs. 26%), and obesity (25% vs. 18.4%) which were significantly higher compared to that seen in the non-RAO cohort. A detailed distribution of demographic characteristics in CVD inpatients by the presence of comorbid RAO is shown in Table 1.

**Table 1:** Differences in demographic characteristics in cerebrovascular diseases inpatients RAO: retinal artery occlusion

Variable	RAO (no), in %	RAO (yes), in %	Total, in %	P-value
Age at admission				
18–35 years	11.4	6.8	11.3	<0.001
36–50 years	23.7	25.6	23.7	
51–65 years	64.9	67.6	65.0	
Sex				
Male	43.8	45.6	43.9	0.146
Female	56.2	54.4	56.1	
Race/ethnicity				
White	54.3	46.8	54.1	<0.001
Black	25.8	30.0	25.9	
Hispanic	12.0	13.8	12.0	
Other	7.9	9.3	7.9	
Comorbidities				
Metastatic cancer	2.3	0.9	2.3	<0.001
Diabetes with complications	18.9	68.5	20.3	<0.001
Hypertension, complicated	26.0	55.3	26.8	<0.001
Obesity	18.4	25.0	18.6	<0.001
Drug abuse	8.7	3.2	8.6	<0.001
Peripheral vascular diseases	10.0	11.8	10.1	0.019

Another very important finding of this study is the effect of geographic region on CVD-related hospitalization with RAO. The demographic differences across the regions were significant, with the highest prevalence in the East North Central (21.2%) and South Atlantic (18.2%), and the lowest in the Mountain (3.8%) and East South Central (3.8%) regions, as shown in Table 2.

**Table 2:** Prevalence of cerebrovascular disease-related hospitalization with retinal artery occlusion according to geographical region in the United States

Geographical region	Prevalence (%)
New England	5.9%
Middle Atlantic	12.1%
East North Central	21.2%
West North Central	8.5%
South Atlantic	18.2%
East South Central	3.8%
West South Central	12.4%
Mountain	3.8%
Pacific	14.1%

There is a statistically significant difference seen in the severity of illness between the cohorts (P<0.001). A higher proportion of the CVD inpatients with RAO had a major loss of functioning (72.1% vs. 58.3% in non-RAO). There existed statistically no significant difference in LOS between the cohorts (P = 0.022), but the total charges per inpatient stay were lower for the RAO cohort ($126,149 vs. $138,703 in non-RAO). A larger proportion of CVD inpatients with RAO were transferred to SNF/ICF (32.4% vs. 24.2%) and home health care (15.9% vs. 11.4%) as compared to the non-RAO cohort. However, 1.2% of in-hospital deaths are seen in CVD inpatients with RAO, which was lower as compared to non-RAO (3.6%) as shown in Table 3.

**Table 3:** Differences in hospitalization outcomes in cerebrovascular diseases inpatients RAO: retinal artery occlusion; LOS: length of stay; SNF: skilled nursing facilities; ICF: intermediate care facilities

Variable	RAO (no)	RAO (yes)	Total	P-value
Severity of illness, in %				
Minor loss of function	6.6	2.1	6.5	<0.001
Moderate loss of function	35.1	25.9	34.8	
Major loss of function	58.3	72.1	58.6	
Other outcomes				
Mean LOS, in days	8.3	9.4	-	0.022
Mean cost, in $	138,703	126,149	-	<0.001
Disposition, in %				
Routine	55.4	47.6	55.2	<0.001
Transfer to short-term hospital	3.6	2.4	3.6	
Transfer to SNF/ICF	24.2	32.4	24.4	
Home health care	11.4	15.9	11.5	
Against medical advice	1.8	0.6	1.7	
Died in hospital	3.6	1.2	3.6	

While studying the effect of various factors on the risk of CVD-related hospitalization in the RAO cohort, it was found that demographics had a statistically non-significant association. The most glaring findings were the effects of comorbidities on the risk of CVD-related hospitalization in the RAO cohort. Diabetes with complications had the most significant impact on the risk of CVD-related hospitalization, which was increased by eight times (OR 7.85, 95% CI 6.98-8.82), followed by hypertension (OR 1.78, 95% CI 1.59-1.98) and PVD (OR 1.21, 95% CI 1.03-1.41). The risk of CVD-related hospitalization was directly related to the increasing severity of illness, with inpatients having major loss of functioning at a higher risk (OR 2.75, 95% CI 1.95-387) as shown in Table 4.

**Table 4:** Risk factors for cerebrovascular disease-related hospitalization in retinal artery occlusion

Variable	Odds ratio	95% Confidence interval		P-value
		Lower limit	Upper limit	
Age at admission				
18–35 years	Reference			
36–50 years	1.06	0.85	1.31	0.617
51–65 years	0.84	0.68	1.03	0.090
Sex				
Male	Reference			
Female	0.99	0.89	1.10	0.876
Race/ethnicity				
White	Reference			
Black	1.05	0.93	1.19	0.440
Hispanic	1.01	0.87	1.19	0.861
Other	1.07	0.89	1.29	0.495
Comorbidities				
None	Reference			
Metastatic cancer	0.49	0.29	0.82	0.007
Diabetes with complications	7.85	6.98	8.82	<0.001
Hypertension, complicated	1.78	1.59	1.98	<0.001
Obesity	0.92	0.81	1.03	0.158
Drug abuse	0.41	0.31	0.54	<0.001
Peripheral vascular diseases	1.21	1.03	1.41	0.020
Severity of illness, in loss of function				
Minor	Reference			
Moderate	1.89	1.33	2.68	<0.001
Major	2.75	1.95	3.87	<0.001

## Discussion

The incidence of stroke in the general population is estimated at 0.03% to 1% [10], whereas it is over 5% in RAO patients [11]. A large cohort study (N = 6628) conducted in Denmark concluded that the incidence of CVD is 5.89% within the first year after an event of RAO [11]. Moreover, studies have shown that in the setting of acute stroke with high-risk factors, the odds of recurrent strokes are associated with retinal vessel changes [12]. Our study demonstrated the prevalence of RAO in CVD-related hospitalizations to be 2.7%. The highest prevalence of CVD hospitalizations in patients with RAO was seen in the East North Central region, followed by the South Atlantic region in the US. The South Atlantic is part of the famously known "stroke belt" due to the prevalence of high-risk factors including hypertension, diabetes, and cigarette smoking [13].

Our study found that patients with RAO were at an increased risk of CVD hospitalization due to comorbid diabetes (risk increased by eight times) and hypertension (risk increased by two times). Diabetes results in macrovascular (like atherosclerosis of major arteries) and microvascular changes (like retinal arteriolar narrowing leading to retinopathy and cerebral small vessel disease) in the vasculature of the whole body [14,15]. Similar vascular changes are caused by hypertension as well. This end-organ damage increases the risk of both RAO and CVD. A meta-analysis by Lau et al. reported that almost a third of patients with CVD had diabetes, and most of the studies included in their systematic review reported high mortality rates, poor neurological and functional outcomes, and longer hospital stays [16]. Management of hyperglycemia in post-stroke patients has been considered a cornerstone of stroke management as chronic hyperglycemia can lead to poorer clinical outcomes [14]. Hypertension not only increases the risk of stroke by remodeling both large and small cerebral blood vessels but also worsens its prognosis by altering the brain’s response to ischemia [17].

RAO has long been called an analogous or marker of CVD for many reasons. For one, both are caused by blockage of blood flow due to emboli formed as a result of systemic atherosclerosis of major blood vessels [18]. Carotid artery plaques are the most common cause of embolism in RAO, and both share the risk factors of atherosclerosis, including hypertension, diabetes, and hyperlipidemia. Since the retina and optic nerve develop as extensions of the brain, they are considered to be brain tissue. The retinal and intracranial circulation share the same origin from the internal carotid artery, which explains the simultaneous emboli to the retina and brain [18,19]. Even the microvasculature of both organs is homologous. These similarities explain the parallel changes in brain vasculature to the changes in retinal vasculature [20].

The relationship between the presence of RAO in CVD inpatients and the severity of illness was found to be statistically significant. CVD inpatients with RAO had higher chances of major loss of function (72% vs. 58%), possibly due to the widespread atherosclerotic disease burden in this cohort. This finding can be further strengthened by the differences found in the adverse disposition of these patients. According to our study, CVD inpatients with RAO needed more extensive care, requiring discharge to skilled nursing homes/intermediate care facilities or enrollment into home health care. The length of inpatient stay was noted to be higher but not statistically significant. However, the mean cost of care was statistically lower in CVD inpatients with RAO compared to CVD inpatients without RAO.

The RAO and CVD relationship has been given immense significance and is an important area of research because the life-threatening nature of CVD in combination with RAO makes it one of the leading causes of death and long-term disability, posing it as a huge public health burden. A study estimated the total cost of CVD treatment in 2016 in the US was $103.5 billion [21]. Of that, 66% accounted for indirect costs from underemployment and premature death, and the age group of 45-64 were the largest consumers of direct costs [21]. As pointed above, larger proportion of inpatients with co-diagnosis of RAO required a high level of care post-discharge and were transferred to nursing facilities and home health care, which might be the increased cost of care. In our study, we found the cost of care for CVD inpatients with RAO was 10% less than those without RAO. Although we could not find anything significant in the literature to compare and study the cause of this difference.

As a limitation of this retrospective study, we could not establish causation. The administrative nature of the NIS data lacks patient-level clinical information; hence, there may be underreporting of comorbidities. The exact time of diagnosis for CVD and RAO was not clarified given the nature of the data set. However, NIS offers large datasets and offers an incomparable population-based perception of disease associations with systematic and temporal factors. Additionally, the information is coded independently by the individual practitioners, so it’s subject to minimal reporting bias.

## Conclusions

The prevalence of RAO in CVD hospitalization was 2.7%, and comorbidities enormously increased the risk of hospitalization in CVD patients with RAO. The risk is increased eight times by diabetes and two times by hypertension. With the rising incidence of stroke, our finding emphasizes the importance of strict management of these comorbidities in patients with CVD. The higher severity of illness in these patients increased the risk of hospitalization and adverse disposition. Moreover, this risk has been found to be highest in certain geographical areas, namely the East North Central Atlantic and South Atlantic regions of the US. Meanwhile, demographics had no significant effect on the risk of hospitalization in these patients. With CVD being a huge public health burden, this data will allow us to devise strategies to timely identify and manage these at-risk patients, which in turn can bring down healthcare costs. This also calls for a collaborative care model to improve the quality of life in these at-risk patients with RAO.
